# School Functioning in Adolescents With Chronic Fatigue Syndrome

**DOI:** 10.3389/fped.2018.00302

**Published:** 2018-10-16

**Authors:** Sarah Jenette Knight, Jennifer Politis, Christine Garnham, Adam Scheinberg, Michelle Anne Tollit

**Affiliations:** ^1^Murdoch Children's Research Institute, Melbourne, VIC, Australia; ^2^The Royal Children's Hospital, Melbourne, VIC, Australia; ^3^Department of Pediatrics, The University of Melbourne, Melbourne, VIC, Australia; ^4^Melbourne Graduate School of Education, The University of Melbourne, Melbourne, VIC, Australia; ^5^Department of Pediatrics, Monash University, Melbourne, VIC, Australia; ^6^The Royal Children's Hospital Education Institute, Parkville VIC Australia, Melbourne, VIC, Australia

**Keywords:** adolescents, chronic fatigue syndrome, school absence, academic performance, school, chronic health condition

## Abstract

**Background:** It is well known that adolescents with chronic fatigue syndrome (CFS) experience greater school absenteeism compared to healthy adolescents. Less is known about other important aspects of school functioning including school participation, school connectedness, and academic performance in students with CFS. The aim of this study was to compare school functioning as a multifaceted construct in adolescents with CFS to healthy adolescent peers. We also explored whether illness factors were associated with school functioning in adolescents with CFS.

**Methods:** Thirty-nine participants with CFS and 28 healthy controls (aged 13–17 years) completed a range of subjective and objective measures of school functioning, as well as measures of fatigue and emotional symptoms.

**Results:** Adolescents with CFS demonstrated significantly higher rates of school absence, as well as poorer school-related quality of life, reduced school participation, poorer connectedness with school, and reduced academic performance. Fatigue severity and emotional symptoms were significantly associated with most aspects of school function.

**Conclusions:** Adolescents with CFS are at increased risk for poor school functioning across a range of indicators which extend beyond school absenteeism.

Adolescent chronic fatigue syndrome (CFS) is a complex condition that is characterized by intense, medically unexplained fatigue together with a range of sleep, pain, cognitive, neuroendocrine, and immune symptoms (1). The estimated incidence of CFS in children and adolescents varies widely (from 0.003 and 2.0%); however, it is consistently found to be more common in females (2, 3). CFS is associated with significant functional disability and this has a considerable impact on emotional, physical, and social functioning (4–9).

Due to the significant functional disability associated with CFS, several studies have associated CFS with high rates of school absence (4, 9–14). The average amount of time away from school for students with CFS has been estimated to be 1 year across their school life (15).

Most studies evaluating school functioning in the context of CFS have been limited by the use of relatively narrow definitions of school functioning, such as defining school functioning solely in terms of school attendance/absence (16). Adolescents with CFS have described difficulties with completing subject requirements and keeping up with academic work (17) and have also reported that their condition impacted on their education or career plans (18). Beyond school attendance, domains of functioning including academic performance, school participation, and school connectedness, have seldom been formally investigated in students with CFS despite their demonstrated links to school success and positive adjustment (16, 19). Taking a broader, more holistic approach to assessing school functioning is crucial in order to more comprehensively understand the impact of CFS and to help inform targeted strategies to optimizing educational outcomes in this vulnerable group (16). There is also limited research directly comparing school functioning in adolescents with CFS with their healthy peers (16). The lack of available normative rates of school absence and other indicators of school functioning makes it difficult to interpret findings and understand the extent and implications of school difficulties for students with CFS. This is in contrast to the larger body of research that has enhanced our understanding of the impact on school functioning in other chronic health conditions, such as childhood cancer, asthma, attention-deficit hyperactivity disorder, gastrointestinal diseases, and chronic pain (20–22).

The relationships between illness factors (e.g., fatigue severity), emotional symptoms, and school functioning in CFS have seldom been a focus of research. In a large sample of patients with CFS, Crawley and Sterne (13) found that children with better physical functioning were more likely to attend school. However, there was no evidence that gender, age, illness duration, anxiety, depression, or pain were associated with school attendance.

To summarize, there is substantial evidence that adolescents with CFS miss large amounts of school; however, our understanding of the impact of CFS on specific aspects of school functioning is limited and requires further exploration. A more thorough understanding of school functioning is an important step toward identifying risk and protective factors associated with school outcomes in the context of CFS. The aim of the current study was to compare adolescents with CFS to healthy peers across multiple aspects of school functioning, including school absence, quality of life in the school setting, school participation, school connectedness, and academic achievement. We also sought to determine whether fatigue severity and emotional symptoms were associated with aspects of school functioning in adolescents with CFS.

## Materials and methods

### Participants

Patients aged between 13 and 17 years with a diagnosis of CFS were recruited from a pediatric tertiary hospital, The Royal Children's Hospital, Melbourne, Australia. To be included in the CFS group, participants required a formal diagnosis of CFS made by a pediatrician specializing in CFS at the tertiary institution. Diagnoses were made via diagnostic interview, laboratory examinations and a medical examination using the Canadian criterion reference (1).

Control participants aged between 13 and 17 years were recruited via convenience sampling (i.e., researchers approached family members, friends, and work colleagues with children in the study age range).

Eligibility of both groups required the ability to speak or read sufficient English to complete the self-report questionnaire and assessments, and at least one parent with sufficient English to complete questionnaires. Exclusion criteria for the CFS group included severe cognitive impairment, learning disability, and/or permanent school absence or home schooling. Participants in the control group were excluded if there was a severe cognitive impairment, neurological disorder, learning disability, chronic health condition, psychiatric diagnosis, and/or permanent school absence or home schooling.

### Measures

#### Demographics and medical information

Basic demographic information (age, gender, ethnicity, and parental education status) were collected via an online questionnaire completed by parents. Parents were asked when their child first started displaying signs of fatigue. Estimated illness duration was calculated in months, from date of initial signs of illness to date of assessment.

#### Estimated intelligence

Level of general intellectual function was estimated using the Wechsler Abbreviated Scale of Intelligence—Second Edition (WASI-II) (23). The WASI-II is a brief standardized measure that provides an estimate of general intellectual ability in 6–89 year olds (Full Scale IQ-2 score or FSIQ-2) across two subtests, including Vocabulary and Matrix Reasoning. The Vocabulary subtest provides a measure of verbal crystallized knowledge. The Matrix Reasoning subtest measures nonverbal fluid abilities. Reliability coefficients for the FSIQ-2 for adolescents range from 0.92 to 0.95, with an average of 0.93 (23).

#### Fatigue symptoms severity

Symptoms of fatigue were measured in the CFS group and control group using The Pediatric Quality of Life Inventory- Multidimensional Fatigue Scale, self-report version (12–18 years; PedsQL MFS) (24, 25). The PedsQL MFS is comprised of 18 items and three dimensions, including; General, Sleep/Rest and Cognitive fatigue, as well as a Total Fatigue score. The 18 items are rated on a five-point scale: “Never,” “Almost Never,” “Sometimes,” “Often,” and “Almost Always.” Respondents were asked how much of a problem each item had been during the past 1 month. For example, “I feel too tired to spend time with my friends”; “I sleep a lot”; “It's hard for me to think quickly.” Items are reverse scored and linearly transformed to a 0–100 scale (0 = 100, 1 = 75, 2 = 50, 3 = 25, 4 = 0), higher scores indicate fewer problems with symptoms of fatigue, lower scores indicate greater problems with symptoms of fatigue. The scale scores of this measure have demonstrated good to excellent patient self-report reliability and validity across a number of pediatric chronic health conditions, including chronic fatigue syndrome (25, 26).

#### Emotional symptoms

Emotional symptoms were measured using the self-report version of the Strengths and Difficulties Questionnaire (SDQ) (27). The SDQ is a 25 item questionnaire that explores common child and adolescent behavioral and emotional problems using a three-point Likert scale (0 = “Not true,” 1 = “Somewhat True,” and 2 = “Certainly True”). A high score on the Emotional Symptoms sub-scale indicates greater problems. The SDQ is one of the most widely used measures in child mental health research and has demonstrated acceptable reliability and content validity (28).

### Measures of school functioning

#### Extra educational supports

Parents of adolescents in both the CFS group and control group were asked the following question in order to obtain data on external educational support: “Does your child receive any extra educational support or modifications to their schooling?” The following categories were provided; “Modified curriculum,” “Reduced work load,” “Visiting teacher service,” “Private tutoring,” “Individual school tutoring or regular individual student/teacher contact,” “Access to distance education,” “Access to distance education as a dual enrolment with their regular school,” “Access to the Program for Students with Disabilities funding (DEECD),” “Other.”

#### School absence

School absence was measured by asking parents of adolescents in the CFS group and control group: “How much school on average has your child missed due to illness or being sick or unwell in the last term?” The degree of absence was indicated using the following response set: None (My child didn't miss any school), About 10% (e.g., one half day per week), About 20% (e.g., 1 day per week), About 30% (e.g., one and a half days per week), About 40% (e.g., 2 days per week), About 50% (e.g., two and a half days per week), About 60% (e.g., 3 days per week), About 70% (e.g., three and a half days per week), About 80% (e.g., 4 days per week), About 90% (e.g., four and a half days per week), 100% (My child did not attend school), Not applicable (N/A).

#### Quality of life in the school setting

Quality of life in the school setting was measured using the 5-item School Functioning subscale of the Pediatric Quality of Life Inventory—self report (PedsQL, V 4.0) (29). The School Functioning scale is comprised of five items, which are rated on a five-point scale in terms of the degree to which the respondent reports having problems with school functioning: “Never,” “Almost Never,” “Sometimes,” “Often,” and “Almost Always.” The PedsQL 4.0 School Functioning scale score has good reliability (α = 0.72) and validity in a range of health conditions, including CFS (25, 29). Items are reverse scored and linearly transformed to a 0–100 scale (0 = 100, 1 = 75, 2 = 50, 3 = 25, 4 = 0). The School Functioning score was calculated by averaging the five school functioning items. Higher scores indicate better overall school functioning. Items on the subscale assess problems regarding “keeping up with school work,” “paying attention in class,” and “forgetting things” in the context of school.

#### School participation

To assess the extent to which participants take part in school activities all participants completed The Child and Adolescent Scale of Participation (CASP) (30), school participation sub-test. The CASP is a reliable and valid measure with high internal consistency (α = 0.96) (30). This measure consists of 20 ordinal-scaled items and four subsections: (1) Home Participation, (2) Community Participation, (3) School Participation, and (4) Home and Community Living Activities. The 20 items are rated on a four-point scale: “Age Expected (Full participation),” “Somewhat Restricted,” “Very Restricted,” and “Unable.” A total school participation score was obtained by averaging the sum of all five school participation items. Higher scores indicate better school participation.

#### School connectedness

To obtain a measure of school connectedness The Psychological Sense of School Membership (PSSM) (31) scale was completed by all participants. This scale measured student's perceptions of belonging, connectedness and psychological engagement in school. The PSSM is a reliable and valid measure with good internal consistency (α = 0.88) (31). The PSSM includes 18 items rated on a five-point Likert scale, ranging from 1 = “Not at all True” to 5 = “Completely true.” Reverse scoring is necessary for five of the items; the scores are then summed into a total score. Higher scores on the PSSM indicate better connectedness and psychological engagement with school.

#### Academic performance

Participants were administered the Wechsler Individual Academic Achievement Test—Australian Abbreviated—Second Edition (WIAT-II-A) (32), to assess their current academic ability. The WIAT-II-A is a reliable and valid, age-standardized measure that assesses the academic achievement of individuals across three subtests: Word Reading, Numeral Operations, and Spelling. Scores from subscales are added to provide an overall academic composite. Standard scores have a mean of 100 and standard deviation of 15. Higher scores indicate better academic functioning.

### Procedure

Ethics approval was obtained from The Royal Children's Hospital's Human Research Ethics Committee (HREC#34060) and written, informed consent was obtained from all participants and their parents. Participants were recruited for this study over a 15 month period (April 2014–July 2015). Families interested in participating in the study were screened over the phone to confirm that they met study criteria. Families were emailed a link to complete the secure online questionnaire. Study data was collected and managed using REDCap electronic data capture tool (33). REDCap is a secure, web-based application designed to support data capture for research studies. Formal assessments using the WASI-II and WIAT-II-A were completed in a quiet room dedicated to such assessments at the hospital.

### Data analysis

All data were analyzed using Stata 14 (34). Preliminary analyses included chi-square, Mann-Whitney U, and independent samples *t*-tests to assess group differences on demographic and illness characteristics. Group differences in reported school supports was also evaluated using chi-square statistics. Effect sizes were also reported. Cohen's d was used for continuous data and Cramer's V for categorical data.

For the first aim, due to group differences in age and estimated intelligence, separate linear regression analyses were conducted to evaluate group differences in school functioning, where each individual school functioning variable was the outcome and “group” (i.e., CFS/control group) was the predictor, adjusting for age and estimated FSIQ. Mean differences (i.e., unstandardized regression coefficients [*b*]) with *p* values were reported. Given the multiple comparisons made in this study, standardized regression coefficients (β) were also evaluated. The standardized regression coefficients were used as measures of the effect size (cutoff points: 0.1 = weak prediction, 0.3 = moderate prediction, 0.5 = strong prediction).

For the second aim, separate linear regression analyses were conducted for the CFS group only to investigate the association between the school functioning variables and illness duration, fatigue, physical health, and emotional symptoms. The *b* values, significance level, coefficient of determination (R2 as a percentage) and β values of each model were reported. Significance for all analyses was determined at an alpha level of 0.05.

## Results

### Sample characteristics

Out of the 78 CFS patients who were invited to participate in the study, 39 consented and participated, constituting a recruitment rate of 50%. Of the families who did not consent (*n* = 39), reasons for non-participation included health reasons, distance from hospital, time, disinterest in study, unable to be contacted, and unable to schedule academic assessment during study period.

The overall sample in this study comprised 67 adolescents (39 CFS, 28 healthy controls). Of the 39 patients in the CFS group, 4 participants did not compete the CASP. There was no missing data in the control group.

On key demographic variables, there were no significant group differences with the exception of small, but statistically significant differences in age (*p* = 0.003) and estimated intelligence (*p* = 0.011) (Table 1). Correspondingly, these variables were used as covariates in further between group analyses. Both groups had a higher preponderance of females. Parental educational status was similar in both groups with the majority of mothers (primary caregiver) reporting a minimum of tertiary education. In the CFS group, the majority reported an infectious illness as a trigger for their child's illness and illness duration varied greatly (11–75 months). As shown in Table 2, compared to the healthy control group, the CFS group were significantly more likely to have a modified curriculum (*p* = 0.001), reduced work load (*p* < 0.001) and access to the visiting teacher service (*p* < 0.001), but not private tutoring, individual school tuition or access to distance education.

**Table 1:** Sample characteristics.

	**CFS group (*n* = 39)**	**Control group (*n* = 28)**	***p*-value, effect size**
Age, M (SD, range)	16.34 (1.15, 13.59–17.93)	15.41 (1.38, 13.0–17.38)	0.003, 0.74
Female sex, % (n)	76.74 (33)	62.07 (18)	0.18, 0.16
Ethnicity, % (n)			0.53, 0.18
Caucasian or European	95.12 (39)	92.86 (26)	
Asian	2.44 (1)	3.57 (1)	
Aboriginal or Torres Strait Islander	2.44 (1)	–	
Other	–	3.57 (1)	
Highest level of education (Mother), % (n)			0.53, 0.21
Did not complete high school	12.2 (5)	3.57 (1)	
Completed high school	4.88 (2)	10.71 (3)	
Some university, TAFE or certification course	21.95 (9)	14.29 (4)	
University degree	39.02 (16)	50.00(14)	
Postgraduate degree	21.95 (9)	21.43 (6)	
Estimated intelligence, M (SD, range)	108.26 (9.69, 88–129)	114.11 (8.38, 89–128)	0.011, 0.64
Illness trigger, % (n)			
Infectious illness	60.4 (17)	–	
Trip or vacation	4.7 (2)	–	
Surgery	2.3 (1)	–	
Stress	20.9 (9)	–	
Other	16.3 (7)	–
No identifiable trigger	11.6 (5)		
Estimated illness duration (months), M(SD, range)	37.96 (17.71, 11–75)	–	

**Table 2:** Extra educational support.

**Extra educational support, % (*n*)**	**CFS group (*n* = 39)**	**Control group (*n* = 28)**	***p*-value, effect size**
Modified curriculum	37.2 (16)	3.5 (1)	0.001, 0.39
Reduced workload	41.9 (18)	3.5 (1)	<0.001, 0.56
Visiting teacher service	11.6 (5)	0	<0.001, 0.56
Private tutoring	14.0 (6)	3.5 (1)	0.14, 0.17
Individual school tuition	14.0 (6)	3.5 (1)	0.14, 0.17
Access to distance education	11.6 (5)	0	0.06, 0.22
Other educational supports	14.0 (6)	6.9 (2)	0.35, 0.11
No educational supports	4.7 (2)	69.0 (20)	<0.001, 0.68

### Group differences in school functioning

The first aim was to investigate group differences in aspects of school functioning between participants with CFS and the healthy control group. As shown in Table 3, the CFS group reported significantly greater school absence, poorer quality of life in the school setting, and reduced participation (all *p* < 0.0001, β > 0.5) compared to the control group. Significantly reduced school connectedness and academic performance were also observed in the CFS group, but with smaller effect (*p* < 0.05, β < 0.3). As expected, participants with CFS reported significantly higher levels of fatigue (*p* < 0.0001, β = 0.80) and emotional symptoms (*p* = 0.015, β = 0.33), compared to healthy controls (Table 3).

**Table 3:** Mean differences between CFS group and controls on school functioning, fatigue and emotional symptoms.

	**CFS group (*n* = 39)**	**Control group (*n* = 28)**	Mean difference^*^ (95% CI)	***p*-value, β**
M (SD, range)				
School absence, %	42.10 (29.75, 0–100)	5 (8.39, 0–30)	−36.7 (−49.74, −23.65)	<0.0001, 0.62
Quality of life in the school setting	37.25 (16.60, 0–70)	77.59 (14.05, 50–100)	38.30 (29.82, 46.78)	<0.0001, 0.76
School participation^†^	81.70 (13.74, 40–100)	98.36 (4.64, 80–100)	15.87 (9.63, 22.12)	<0.0001, 0.58
School connectedness	67.0 (13.10, 38–87)	73.59 (11.03, 44–90)	6.98 (.10, 13.86)	0.047, 0.27
Academic performance	101.11 (12.38, 70–125)	111.81 (11.58, 82–129)	5.98 (0.78, 11.17)	0.025, 0.23
Fatigue	36.01 (15.0, 11.11–77.78)	73.23 (12.20, 41.67–95.83)	37.99 (30.13, 45.84)	<0.0001, 0.80
Emotional symptoms	4.29 (2.84, 0–9)	2.34 (1.80, 0–7)	−1.70 (−3.05, −0.35)	0.015, 0.33

* All variables are adjusted for age and estimated FSIQ; CFS, chronic fatigue syndrome.

† *N_CFS_ = 35, N_Control_ = 28*.

### Influence of fatigue and emotional symptoms on school functioning in CFS group

The second aim was to investigate the relationship between fatigue and emotional symptoms, and school functioning within the CFS group. Regression results are shown in Table 4. Fatigue levels were strongly associated with all aspects of school functioning (all *p* < 0.001, β = 0.53–0.69) with the exception of academic performance. Fatigue levels were able to explain a substantial amount of the variance in school absence, quality of life in the school setting, school participation and school connectedness (R^2^ = 28–48%). Emotional symptoms were significantly associated with quality of life in the school setting (*p* < 0.001, β = 0.51), school participation (*p* = 0.01, β = 0.18), and school connectedness *p* < 0.0001, β = 0.55), but not school absence or academic performance. Overall, the amount of variance explained by emotional symptoms appeared slightly lower compared to fatigue levels (R^2^ = 18–31%). All findings for the second aim held when re-run with age and estimated FSIQ as covariates.

**Table 4:** Association of fatigue and emotional symptoms with school functioning in CFS group.

	**Fatigue**		**Emotional symptoms**	
	***b*, *R^2^* (95% CI)**	***p*-value, β**	***b*, *R^2^* (95% CI)**	***p*-value, β**
School absence, %	−0.10, 0.28 (−0.16, −0.49)	<0.001, 0.53	0.29, 0.08 (−0.04, 0.63)	0.09, 0.29
Quality of life in the school setting	0.76, 0.48 (0.50, 1.03)	<0.0001, 0.69	−2.99, 0.26 (−4.70, −1.29)	<0.001, 0.51
School participation^†^	0.58, 0.45 (0.35–0.81)	<0.0001, 0.67	−2.08, 0.18 (−3.66, −0.50)	0.01, 0.18
School connectedness	0.51, 0.35 (0.28, 0.75)	<0.0001, 0.59	−2.53, 0.31 (−3.81, −1.24)	<0.0001, 0.55
Academic performance	0.04, 0.00 (−0.24, 0.32)	0.76, 0.05	−0.30, 0.00 (−1.88, 1.27)	0.70, 0.07

CFS, chronic fatigue syndrome.

† *N_CFS_ = 35*.

## Discussion

The aim of this study was to extend our knowledge about school functioning in adolescents with CFS. Overall, compared to healthy adolescents, school functioning was compromised for adolescents with CFS across several specific domains (including school absence, quality of life in the school setting, school participation, school connectedness, and academic performance). Further, greater severity of fatigue in adolescents with CFS was associated with lower levels of school attendance, quality of life in the school setting, participation and connectedness, but not academic performance. Emotional symptoms were significantly associated with quality of life in the school setting, participation and connectedness, but not school absence or academic performance.

### Group differences in school functioning

Current study findings suggest that adolescents with CFS receive significantly more external education support or modifications to the curriculum compared to healthy controls. Further, the CFS group reported poorer school functioning across all areas compared to the control group. As expected and consistent with previous studies reporting high rates of school absence (12–15, 18, 35–37), school absence rates due to illness for the CFS group were substantially higher than in the control group. On average, the control group missed less than one half a day per week over the last term due to illness, as opposed to the CFS group who were absent for, on average, 40% of the school term due to illness. These findings suggest that students with CFS are missing substantial amounts of school and report restrictions to school participation. Not only do these limitations have implications for the development of core academic skills, but such functional impairment also has potential to impact on social competencies which are integral for healthy adolescent development. Schools need to work toward supporting these students to ensure they remain connected and engaged with school when their medical condition impacts on their ability to physically attend school on a full-time basis.

When controlling for age and intelligence, the adolescents with CFS showed significantly reduced academic functioning compared to the healthy control sample, overall. Of note, both groups displayed high SES (based on educational status of primary caregiver) as well as above average intelligence. While the mean score for academic functioning for the CFS group fell within the average range in the context of normative population expectations, given the relatively high SES of the group and the overall above average level of intellectual functioning, these results suggest that compared to healthy controls, these adolescents may not be performing to their full potential academically (38). These are valuable findings as to the author's knowledge this is the first analysis of academic performance in adolescents with CFS using a standardized achievement test. Other studies exploring school performance in CFS using patient self-reports (17, 39) have also reported concerns regarding academic achievement.

### Relationships between fatigue and emotional symptoms, and school functioning in the CFS group

As expected, greater fatigue severity was associated with higher school absence rates due to illness, as well as poorer school-related quality of life, lower school participation and connectedness. This is commensurate with findings from previous research whereby severity of physical health, were associated with school attendance (18, 40). Further, while greater emotional symptoms did not appear to relate to school absence, they were associated with poorer school-related quality of life, lower school participation and connectedness, suggesting that while school attendance may not be additionally affected for adolescents with CFS with more significant emotional symptoms, other less obvious aspects of school functioning may be further affected.

No significant associations were observed between fatigue and emotional symptoms, and academic functioning. The explanation for this discrepancy is unclear; although it is possible that more complex academic skills not assessed in the current study (e.g., comprehension, written expression) could be affected by these factors and further research into this area is warranted.

### Strengths and limitations

To the author's knowledge, this is the first study to comprehensively compare multiple aspects of school functioning in adolescents with a formal diagnosis of CFS with a control group of healthy adolescents. This study has several strengths including the use of a control group, as an indication of how this clinic group is functioning at school compared to their healthy peers.

There are limitations to the study that should be considered when interpreting results. Foremost is the employment of a small sample size in both groups. It is acknowledged that this has implications in terms of statistical power; however, given the uniqueness of such a study in the CFS literature the findings of the current study are of importance. These results should be replicated in future studies that employ a larger sample to confirm current study findings.

This study investigated school functioning from the perspective of the adolescent. Future research could incorporate reports from other informants about school functioning (e.g., parents and teachers) to obtain a more thorough understanding of school functioning. Discussion is also warranted around the representativeness of the CFS sample. Importantly, this study employed a strict diagnostic criterion, the Pediatric Canadian Criterion reference guidelines (1). While the sample characteristics (e.g., age, gender, and SES) are similar to what has been found in epidemiological studies of adolescents with CFS (3, 4, 12, 41, 42), it should be acknowledged that the adolescents participating in this study were recruited from a tertiary hospital and were required to attend the hospital in person for the academic assessment. Therefore, they may not be representative of all adolescents with CFS in the community (e.g., adolescents less severely affected may be managed in primary care and adolescents more severely affected by the illness may not have been well enough to attend the hospital for assessment).

Lastly, the cross-sectional nature of analysis employed in the study prevents exploration of the trajectory of school functioning over time as well as factors or mediators that might influence change in school functioning over time. To expound these questions, future studies could incorporate longitudinal methods to follow the course of CFS and school functioning over time. Despite the present study containing a number of limitations requiring consideration, given the novelty of this research, the study provides a large contribution to our preliminary understanding of school functioning in adolescents with CFS and highlights directions for future research. Future studies should consider investigating academic performance beyond basic reading, spelling and mathematics, as well as the relative contributions of factors such as absenteeism, fatigue, cognitive difficulties, and emotional symptoms, to academic performance.

## Conclusions

The findings from this study indicate that school functioning in adolescents with CFS is significantly poorer than that of healthy adolescents. This study highlights that expanding the indicators of school functioning beyond school absenteeism in adolescents with CFS provides a more comprehensive picture of school functioning that is likely useful in both research and school contexts. School absence due to illness does not accurately capture compromised school functioning in adolescents with CFS, and instead, more sensitive and specific domains such as those employed in the current study should be considered. This study demonstrated that in addition to increased school absence, CFS is also associated with poorer school-related quality of life, school participation, connectedness with school, and academic achievement when compared to healthy adolescent peers. School is the principle location for the development of not only academic skills, but also cognitive, social, and community-related skills during childhood and adolescence. Therefore, the impact that CFS has on school functioning may place these adolescents at a heightened risk of long-term maladjustment across a range of key developmental areas.

### Implications for school health

The findings from the current study have implications for the school health of adolescents with CFS. From a school health perspective, the findings support the early monitoring and careful analyses of specific aspects of school functioning in adolescents diagnosed with CFS, which may in turn help inform targeted intervention programs, designed to minimize the long term impact of poor school functioning. Aspects of school functioning should be screened by professionals working with students with CFS in schools, and frequent liaison between health professionals and school staff is likely to be beneficial. Given the significant impact on school functioning for adolescents with CFS noted by the current study, school staff should be provided with professional development aimed at increasing their understanding of CFS and how it can impact on the school functioning of students. Given the unique needs of each adolescent, as well as vast differences across school settings, tailored and individualized school planning that addresses not only school attendance, but also strategies to minimize the impact of the illness on school-related quality of life, school participation, school connectedness, and academic outcomes, will be crucial.

## Author contributions

SK and MT lead the study design, data analysis and completed the first draft of the manuscript. They also supervised data collection. CG and JP completed data collection and contributed to data analysis and writing of the manuscript draft. AS contributed to study design and reviewed and contributed to manuscript drafts. All authors approved final version of the manuscript prior to submission.

### Conflict of interest statement

The authors declare that the research was conducted in the absence of any commercial or financial relationships that could be construed as a potential conflict of interest.

## References

[1] Jason LA Bell DS Rowe K Van Hoof ELS Jordan K Lapp C A pediatric case definition for myalgic encephalomyalitis and chronic fatigue syndrome. J Chron Fat Synd. (2006) 13:1–44. 10.1300/J092v13n02_01

[2] Nijhof SL Maijer K Bleijenberg G Uiterwaal C Kimpen J van de Putte EM. Adolescent chronic fatigue syndrome: prevalence, incidence, and morbidity. Pediatrics (2011) 127:e1169–75. 10.1542/peds.2010-1147 21502228

[3] Rimes KA Goodman R Hotopf M Wessely S Meltzer H Chalder T. Incidence, prognosis, and risk factors for fatigue and chronic fatigue syndrome in adolescents: a prospective community study. Pediatrics (2007) 119:e603–9. 10.1542/peds.2006-2231 17332180

[4] Crawley E Hughes R Northstone K Tilling K Emond A Sterne JAC. Chronic disabling fatigue at age 13 and association with family adversity. Pediatrics (2012) 130:e71–9. 10.1542/peds.2011-2587 22711716

[5] Crawley E Hunt L Stallard P. Anxiety in children with CFS/ME. Eur Child Adolesc Psychiatry (2009) 18:683–9. 10.1007/s00787-009-0029-4 19452195

[6] Davies S Crawley E. Chronic fatigue syndrome in children aged 11 years old and younger. Arch Dis Childhood (2008) 93:419–21. 10.1136/adc.2007.126649 18192312

[7] Garralda ME Rangel L. Impairment and coping in children and adolescents with chronic fatigue syndrome: a comparative study with other paediatric disorders. J Child Psychol Psychiatry Allied Discipli. (2004) 45:543–52. 10.1111/j.1469-7610.2004.00244.x 15055373

[8] Dickson A Toft A O'Carroll RE. Neuropsychological functioning, illness perception, mood and quality of life in chronic fatigue syndrome, autoimmune thyroid disease and healthy participants. Psychol Med. (2009) 39:1567–76. 10.1017/S0033291708004960 19144216

[9] Kennedy G Underwood C Belch JJ. Physical and functional impact of chronic fatigue syndrome/myalgic encephalomyelitis in childhood. Pediatrics (2010) 125:e1324–30. 10.1542/peds.2009-2644 20478937

[10] Bell DS Jordan K Robinson M. Thirteen-year follow-up of children and adolescents with chronic fatigue syndrome. Pediatrics (2001) 107:994–8. 10.1542/peds.107.5.994 11331676

[11] Bould H Collin SM Lewis G Rimes K Crawley E. Depression in paediatric chronic fatigue syndrome. Arch Dis Child. (2013) 98:425–8. 10.1136/archdischild-2012-303396 23619200

[12] Crawley E Emond A Sterne J. Unidentified chronic fatigue syndrome/myalgic encephalomyelitis (CFS/ME) is a major cause of school absence: surveillance outcomes from school-based clinics. BMJ Open. (2011) 1:e000252. 10.1136/bmjopen-2011-000252 22155938 PMC3244656

[13] Crawley E Sterne JA. Association between school absence and physical function in paediatric chronic fatigue syndrome/myalgic encephalopathy. Arch Dis Child. (2009) 94:752–6. 10.1136/adc.2008.143537 19001477

[14] Knight SJ Harvey A Lubitz L Rowe K Reveley C Veit F. Paediatric chronic fatigue syndrome: Complex presentations and protracted time to diagnosis. J Paediatr Child Health (2013) 49:919–24. 10.1111/jpc.12425 24251657

[15] Rangel L Garralda ME Levin M Roberts H. The course of severe chronic fatigue syndrome in childhood. J R Soc Med. (2000) 93:129–34. 10.1177/014107680009300306 10741312 PMC1297949

[16] Tollit M Politis J Knight S. Measuring school functioning in students with chronic fatigue syndrome: a systematic review. J School Health (2018) 88:74–89. 10.1111/josh.12580 29224219

[17] Van Hoof E De Becker P Lapp CW De Meirleir K How do adolescents with chronic fatigue syndrome perceive their social environment? a quantitative study. Bull IACFS/ME (2009) 17:16–31. Available online at: http://www.iacfsme.org/Portals/0/pdf/Van%20Hoof%20vol17%20n1.pdf

[18] Sankey A Hill CM Brown J Quinn L Fletcher A. A follow-up study of chronic fatigue syndrome in children and adolescents: symptom persistence and school absenteeism. Clin Child Psychol Psychiatr. (2006) 11:126–38. 10.1177/1359104506059133 17087490

[19] Cole DA Maxwell SE Martin JM Peeke LG Seroczynski AD Tram JM. The development of multiple domains of child and adolescent self-concept: a cohort sequential longitudinal design. Child Dev. (2001) 72:1723–46. 10.1111/1467-8624.00375 11768142

[20] DuPaul GJ Morgan PL Farkas G Hillemeier MM Maczuga S. Eight-Year latent class trajectories of academic and social functioning in children with attention-deficit/hyperactivity disorder. J Abnormal Child Psychol. (2018) 46:979–92. 10.1007/s10802-017-0344-z 28913744

[21] Lum A Wakefield CE Donnan B Burns MA Fardell JE Marshall GM. Understanding the school experiences of children and adolescents with serious chronic illness: a systematic meta-review. Child (2017) 43:645–62. 10.1111/cch.12475 28543609

[22] Gorodzinsky AY Hainsworth KR Weisman SJ. School functioning and chronic pain: a review of methods and measures. J Pediatr Psychol. (2011) 36:991–1002. 10.1093/jpepsy/jsr038 21745810

[23] Wechsler D Wechsler Abbreviated Scale of Intelligence (WASI-II). 2nd Edn San Antonio, TX: The Psychological Corporation (2011).

[24] Varni JW Burwinkle TM Szer IS The PedsQL Multidimensional Fatigue Scale in pediatric rheumatology: reliability and validity. J Rheumatol. (2004) 3112:2494–500. 15570657

[25] Knight SJ Harvey A Hennel S Lubitz L Rowe K Reveley C Measuring quality of life and fatigue in adolescents with chronic fatigue syndrome: estimates of feasibility, internal consistency and parent–adolescent agreement of the PedsQLTM. Fatigue (2015) 3:220–34. 10.1080/21641846.2015.1090816

[26] Crichton A Knight SJ Oakley E Babl F Anderson V. Fatigue in child chronic health conditions: a systematic review of assessment instruments. Pediatrics (2015) 135: e1015–31. 10.1542/peds.2014-2440 25802352

[27] Goodman R. The strengths and difficulties questionnaire: a research note. J Child Psychol Psychiatry (1997) 38:581–6. 10.1111/j.1469-7610.1997.tb01545.x 9255702

[28] Vostanis P. Strengths and difficulties questionnaire: research and clinical applications. Curr Opin Psychiatry (2006) 19:367–72. 10.1097/01.yco.0000228755.72366.05 16721165

[29] Varni JW Seid M Kurtin PS PedsQL™ 4.0: reliability and validity of the pediatric quality of life inventory™ version 4.0 generic core scales in healthy and patient populations. Med Care (2001) 39:800–12. 10.1097/00005650-200108000-00006 11468499

[30] Bedell G. Further validation of the child and adolescent scale of participation (CASP). Dev Neurorehabil. (2009) 12:342–51. 10.3109/17518420903087277 20477563

[31] Goodenow C The psychological sense of school membership among adolescents: scale development and educational correlates. Psychol Schools (1993) 30:79–90.

[32] Wechsler D Wechsler Individual Achievement Test-Australian Abbreviated (WIAT-II Abbreviated). 2nd Ed Sydney: Pearson Clinical (2007).

[33] Harris PA Taylor R Thielke R Payne J Gonzalez N Conde JG. Research electronic data capture (REDCap) - a metadata-driven methodology and workflow process for providing translational research informatics support. J Biomed Inform. (2009) 42:377–81. 10.1016/j.jbi.2008.08.010 18929686 PMC2700030

[34] StataCorp Stata Statistical Software: Release 14. College Station, TX: StataCorp LP (2015).

[35] Crawley E Collin SM White PD Rimes K Sterne JA May MT. Treatment outcome in adults with chronic fatigue syndrome: a prospective study in England based on the CFS/ME National outcomes database. QJM (2013) 106:567. 10.1093/qjmed/hct122 23538643 PMC3665909

[36] Lim A Lubitz L. Chronic fatigue syndrome: successful outcome of an intensive inpatient programme. J Paediatr Child Health (2002) 38:295–9. 10.1046/j.1440-1754.2002.00786.x 12047700

[37] van Geelen SM Bakker RJ Kuis W van de Putte EM Adolescent chronic fatigue syndrome: a follow-up study. Arch Pediatr Adolesc Med. (2010) 164:810–4. 10.1001/archpediatrics.2010.145 20819962

[38] Sirin SR Rogers-Sirin L Components of school engagement among African American adolescents. Appl Dev Sci. (2005) 9:5–13. 10.1207/s1532480xads0901_2

[39] Carter BD Edwards JF Kronenberger WG Michalczyk L Marshall GS. Case control study of chronic fatigue in pediatric patients. Pediatrics (1995) 95:179–86. 7838632

[40] Nagane M. Relationship of subjective chronic fatigue to academic performance. Psychol Rep. (2004) 95:48–52. 10.2466/pr0.95.1.48-52 15460357

[41] Farmer A Fowler T Scourfield J Thapar A. Prevalence of chronic disabling fatigue in children and adolescents. Br J Psychiatry (2004) 184:477–81. 10.1192/bjp.184.6.477 15172940

[42] Lloyd AR Hickie I Boughton CR Spencer O Wakefield D. Prevalence of chronic fatigue syndrome in an Australian population. Med J Australia (1990) 153:522–8. 2233474 10.5694/j.1326-5377.1990.tb126191.x

